# Identification of 14-3-3 Family in Common Bean and Their Response to Abiotic Stress

**DOI:** 10.1371/journal.pone.0143280

**Published:** 2015-11-23

**Authors:** Ruihua Li, Xiaotong Jiang, Donghao Jin, Sangeeta Dhaubhadel, Shaomin Bian, Xuyan Li

**Affiliations:** 1 College of Plant Science, Jilin University, Changchun, China; 2 Agriculture and Agri-Food Canada, Southern Crop Protection and Food Research Centre, London, Ontario, Canada; Chinese Academy of Sciences, CHINA

## Abstract

14-3-3s are a class of conserved regulatory proteins ubiquitously found in eukaryotes, which play important roles in a variety of cellular processes including response to diverse stresses. Although much has been learned about 14-3-3s in several plant species, it remains unknown in common bean. In this study, 9 common bean 14-3-3s (PvGF14s) were identified by exhaustive data mining against the publicly available common bean genomic database. A phylogenetic analysis revealed that each predicted PvGF14 was clustered with two GmSGF14 paralogs from soybean. Both epsilon-like and non-epsilon classes of PvGF14s were found in common bean, and the *PvGF14s* belonging to each class exhibited similar gene structure. Among 9 *PvGF14s*, only 8 are transcribed in common bean. Expression patterns of *PvGF14s* varied depending on tissue type, developmental stage and exposure of plants to stress. A protein-protein interaction study revealed that *PvGF14a* forms dimer with itself and with other PvGF14 isoforms. This study provides a first comprehensive look at common bean 14-3-3 proteins, a family of proteins with diverse functions in many cellular processes, especially in response to stresses.

## Introduction

14-3-3 proteins are a group of conserved regulatory molecules that ubiquitously exist in all eukaryotes. Generally, 14-3-3 proteins act as homo- or heterodimers to function through their ability to bind with their phosphorylated protein clients. This process results in alteration in stability, activity, intracellular localization or interaction capability of their client proteins [1–3]. It has been demonstrated that 14-3-3 proteins are able to recognize highly conserved binding motif within their client protein. So far, three canonical motifs have been defined for 14-3-3 binding such as (R/K)SX(S/T)^P^XP, (R/R)XΦX(S/T)^P^XP and (S/T)^P^X_1-2_-COOH [4], where X,Φ and (S/T)^P^ indicate any amino acid, aromatic/aliphatic amino acid, and serine/threonine that could be potentially phosphorylated, respectively. Nevertheless, 14-3-3s can also bind some protein clients by means of noncanonical or phosphorylation-independent motifs such as WLDLE and GHSL [5,6].

Plant 14-3-3 proteins were identified concurrently from *Arabidopsis thaliana*, *Hordeumvulgare*, *Spinaceaoleracea*and *Oenotherahookeri* [7–9]. Since then, many 14-3-3s have been isolated and characterized in several other plant species [10–17]. To date, many efforts have been made to elucidate the roles of 14-3-3s in plant development and response to abiotic stresses [18–23]. Over-expression or silencing of *14-3-3s* influenced stress tolerance in plants. For example, over-expression of Arabidopsis *AtGF14λ* increased drought tolerance in cotton [24], whereas silencing of *AtGF14μ* in Arabidopsis promoted drought tolerance [25]. Similarly, over-expression of *TOMATO 14-3-3 PROTEIN 4* (*TFT4*) in Arabidopsis increased alkaline stress response [26], while the knock-out of *RCI1A/AtGF14ψ* enhanced the constitutive freezing tolerance [27]. Additionally, 14-3-3s themselves can be affected by abiotic stresses. For instance, transcriptional accumulations of *14-3-3s* were altered by cold, heat, drought, salinity and nutrition deficiency [27–31]. 14-3-3s also interact with components of stress signaling pathways such as ABA-responsive element binding factors, involved in ABA-dependent signaling pathway under salinity stresses [32], H^+^-ATPase, creating gradient for stomatal opening [33], SALT OVERLY SENSITIVE 2 (SOS2) that mediates intracellular sodium ion homeostasis and salt tolerance [34].

Compared to other organisms, plants contain a large number of 14-3-3 isoforms. For example, there are 13 14-3-3 protein isoforms in Arabidopsis [35], 8 in rice [16], 16 in soybean [14], 8 in foxtail millet [36] and 10 in rubber [15]. These isoforms are encoded by multi-gene family with small difference in sequence. However, emerging evidences indicated that 14-3-3s exert their regulatory functions in an isoform-specific manner. It has been demonstrated that 14-3-3 isoforms displayed differential subcellular localization, distinct tissue-specific and/or inducible expression [14,15,26,37,38], which implied their specific interactions with cellular clients during developmental processes or in response to diverse stresses. For instance, soybean 14-3-3 isoforms showed different binding affinity to GmMYB176 (an isoflavonoid regulator) [19], while rice 14-3-3 isoforms displayed differential binding specificity towards ACC synthase [39]. Evidently, 14-3-3 isoforms play important roles in determining complexity and specificity of biological functions in plants. Thus, addressing the implications of 14-3-3 family diversity becomes an important step towards elucidating their roles in plant developmental processes and/or resistance to stresses.

Common bean (*Phaseolus vulgaris* L.) is one of the most important crop legumes worldwide. It is a diploid species with 11 chromosomes (2n = 2x = 22) [40], and a genome size of 473 Mb [41]. Although much has been learned about 14-3-3s in several plant species, no 14-3-3 has been identified in common bean. Availability of the whole genome sequence of common bean facilitates to systematically analyze gene family members and their possible roles in common bean. In this study, data mining was conducted against publicly available common bean genomic database, and a total of 9 14-3-3s (*PvGF14s*) were identified. The PvGF14 isoforms showed high sequence conservation with SGF14s from soybean. Furthermore, *PvGF14s* displayed tissue-specific expression patterns, and their transcriptional activities were altered when subjected to cold, drought and salinity stress. These findings provide a foundation for elucidating the roles of *PvGF14s* in common bean during development or in response to abiotic stress.

## Materials and Methods

### Plant materials and treatments

Common bean (*Phaseolus vulgaris* L.) cv Dongbeixiaoyoudou is a local cultivar in the northeast of China. Plants (Dongbeixiaoyoudou) were grown at experimental station in Jilin University (Changchun, Jilin Province, China), in 2013, and seeds were collected for the following experiments.

Seeds of common bean cultivar "Dongbeixiaoyoudou" were surfacesterilized by using 10% (w/v) sodium hypochlorite for 20 min, and then washed thoroughly with sterile distilled water. These sterilized seeds were allowed to germinate in 150mm diameter plate with wet filter paper under sterile conditions. Subsequently, six well-germinated seeds were chosen and sown on each pot filled with 65g vermiculite. All the seedlings were grown under a 14 h light and 10 h dark photoperiod at 25°C (light) and 20°C (dark) in a chamber and regularly watered with Hoagland liquid medium. Ten-day-old seedlings were subjected to the following treatments and six pots of seedlings were used for each treatment: (1) For cold stress, seedlings were transferred to 4°C and samples were collected at 0, 1, 3, 6, 12 and 24h after cold treatment; (2)For drought stress, water supply was withheld and samples were collected at 0, 1, 3, 5, 7 and 9 days of water stress; (3) For salinity stress, 200 mM NaCl solution was applied to seedlings and samples were collected at 0, 3, 6, 12, 33, 48 and 72h after salt treatment. The above-ground parts were collected and frozen in liquid nitrogen and stored at -80°C.

### Identification of PvGF14s in common bean

The known 14-3-3 protein sequences from soybean, Arabidopsis and rice were obtained from NCBI database, which were used as queries to conduct BLAST search against the public genomic database (http://phytozome.jgi.doe.gov/pz/portal.html). The accession numbers of 14-3-3 proteins from Arabidopsis, soybean and rice were listed in S1 Table. Additionally, to identify all PvGF14s, a key word search using the word "14-3-3" was conducted against the common bean whole genome database (http://phytozome.jgi.doe.gov/pz/portal.html#!search?show=KEYWORD&method=Org_Pvulgaris). All the putative PvGF14s were searched for 14-3-3-specific domain and signature using PROSITE (http://prosite.expasy.org/), Pfam (http://pfam.xfam.org/search) and SMART (http://smart.embl-heidelberg.de/) programs. Their sub-cellular localization was predicted using PSORT algorithms with default parameters (http://www.psort.org/).

### Multiple sequence alignment, phylogenetic tree construction and gene structure

Multiple sequence alignment of all putative PvGF14 proteins were performed by Clustal X, and phylogenetic trees were constructed by the Neighbor-joining (NJ) method using MEGA5 software [42]. Bootstrap values were calculated using 1000 replicates. Gene structures of *PvGF14s* were built using SIM4 (http://pbil.univ-lyon1.fr/members/duret/cours/inserm210604/exercise4/sim4.html).

### Calculation of Ka/Ks values

The DnaSP program version 5.10.1 was used to calculate the ratios of non-synonymous (Ka) versus synonymous (Ks) substitution rate (Ka/Ks) for orthologous gene pairs of 14-3-3s [43]. Generally, Ka/Ks = 1 refers to neutral selection, Ka/Ks >1 refers to positive selection to accelerate evolution, and Ka/Ks <1 refers to purifying selection during evolution [44].

### Chromosomal localization and gene duplication

To determine the location of putative *PvGF14s* in common bean chromosomes, coordinate of individual gene and chromosome length were obtained from Phytozome database. *PvGF14s* in duplicated genomic regions and Ka/Ks values for each duplicated *PvGF14* were retrieved from batch download option of Plant Genome Duplication Database (http://chibba.agtec.uga.edu/). Tandem duplications were defined as two paralogs separated by less than five genes in the same chromosome [45], while segmental duplications referred to those homologous genes distributed on duplicated chromosomal blocks from the same genome lineage.

### Expression analysis of *PvGF14s*

Total RNA was isolated from common bean tissues using RNAprep Pure Plant Kit (Tiangen Inc, China), according to the manufacture’s instruction followed by RNase-Free DNase I (NEB Inc, New England) treatment to remove DNA. Total RNA (2μg) was used to synthesize first-strand cDNA using pRimeScript RT reagent kit with gDNA Eraser (Takara Inc, Japan). RT-PCR was performed by using the *PvGF14* gene-specific primers (S2 Table). qPCR was conducted using ABI7500 real-time PCR detection system and SYBR Premix Ex Taq (TakaraInc, Japan). Data were analyzed by ABI7500 software v.2.0.6, using *ACTIN11* as the internal reference. The expression levels of the controls for each type of stress treatments were set as 1, and relative expression level of each *PvGF14* for each treatment was normalized accordingly. The primer sequences used in the study are listed in S3 Table. Statistical significance of the data was analyzed by one-way ANOVA with LSD test, and *p* value < 0.05 was considered to be statistically significant.

To analyze the expressions of *PvGF14s* in different tissues, fragments per kilobase of transcript per million mapped reads (FPKM) values for each *PvGF14* were extracted from Phytozome database by tracking common bean gene-level expression (http://www.phytozome.net). The heatmap for *PvGF14*genes was generated in R using the heatmap.2 function from the gplots CRAN library (http://CRAN.R-project.org/package=gplots). To confirm the RNA-seq data from the public database, RT-PCR was performed by using the *PvGF14* gene-specific primers (S3 Table). For promoter analysis, 1500 bp upstream region of the transcriptional start site of each *PvGF14* gene were analyzed in PlantCARE database (http://bioinformatics.psb.ugent.be/webtools/plantcare/html/).

### Yeast two-hybrid assay

To conduct yeast two-hybrid (Y2H) assay, the gateway-compatible vectors pGBKT7-DEST and pGADT7-DEST were utilized to prepare the bait and the prey, respectively [46]. Full-length *PvGF14a* cDNA was cloned into pGBKT7-DEST as a bait and self-activation was checked. The full-length cDNAs of all *PvGF14s* except *PvGF14q* were cloned separately into the pGADT7-DEST vector as preys. The bait vector and each of the prey vectors containing *PvGF14* gene were co-transformed into yeast strain AH109, and grown on synthetic defined (SD)/-Leu/-Trp selective agar medium. Selected individual yeast transformants were grown on liquid medium, and 5 μL of yeast suspension culture with a series of 10X dilutions was spotted onto SD/-Leu/-Trp and SD/-Ade/-His/-Leu/-Trp plates, and grown for 5 days at 30°C. Empty vectors were co-transformed as negative controls.

## Results

### Identification of common bean 14-3-3 gene family

**Fig 1:**
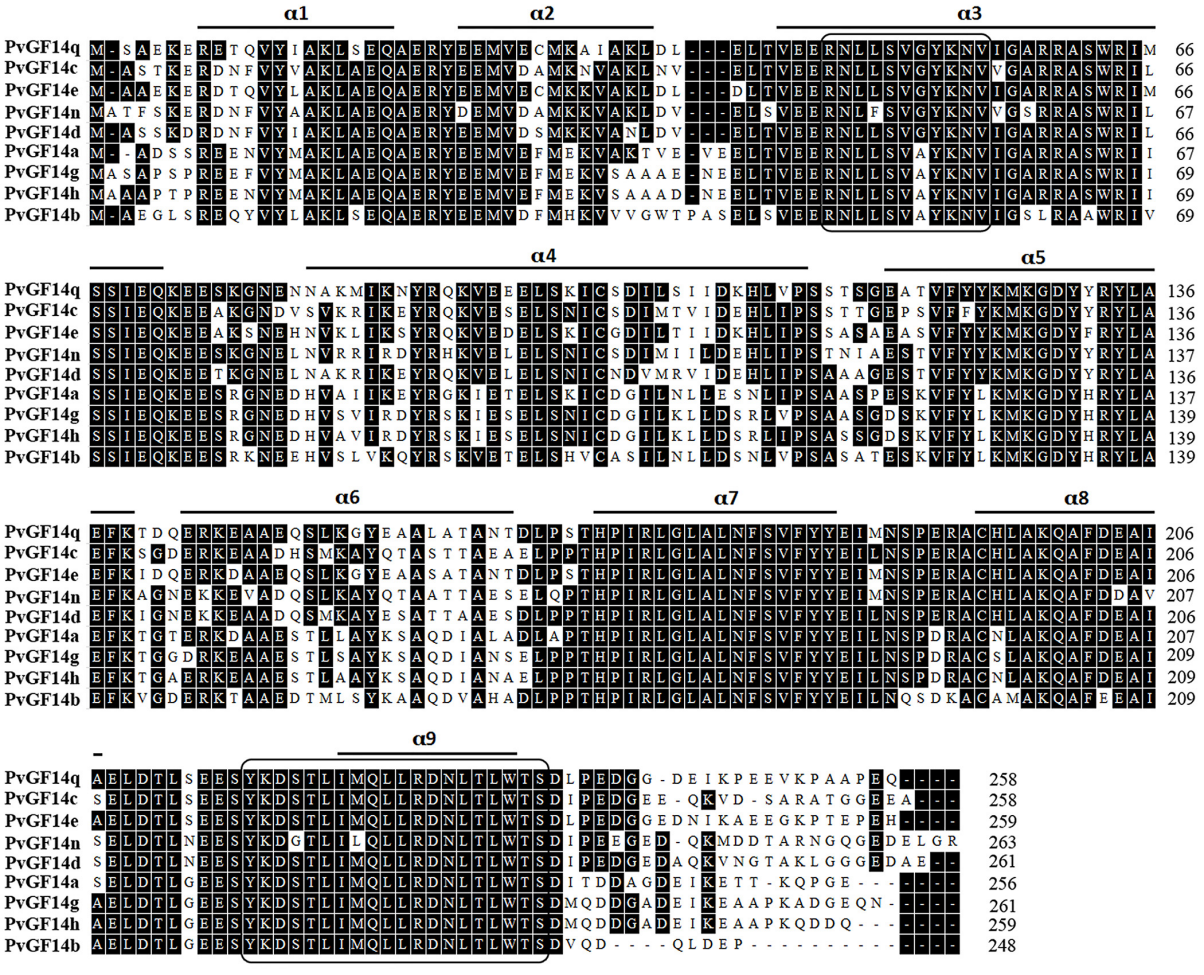
Sequence alignment of candidate PvGF14 proteins. Identical amino acid residues are shown in black. The α-helixs of PvGF14s are shown as a line and 14-3-3 signatures of PvGF14s are shown in rectangular boxes.

To identify 14-3-3 protein genes in common bean, 14-3-3 protein sequences from soybean, Arabidopsis and rice were used as queries to conduct BLAST search against the common bean genomic database (http://www.phytozome.net/). In addition, a key word search using the word ‘14-3-3’ was also conducted against the above database. This process identified a total of 9 putative 14-3-3 genes (*PvGF14a-PvGF14e*, *PvGF14g*, *PvGF14h*, *PvGF14n* and *PvGF14q*), which were named in term of 14-3-3 nomenclature in soybean. Table 1 provides detail information on all putative *PvGF14s*. The deduced 14-3-3 proteins contain 248 to 263 amino acids residues with the calculated molecular weights from 28.19 to 30.21kDa, and the estimated isoelectric points from 4.56 to 4.85. As shown in Fig 1, all the predicted PvGF14s contain a 14-3-3 conserved domain featured by one or two 14-3-3 protein signatures, and they were predicted to localize in cytoplasm, peroxisome, chloroplast and/or mitochondria (Table 1).

**Table 1:** Characteristics of *PvGF14s* genes in common bean.

Gene name	Locus name	Chromosomal location^a^	Protein			Predicted subcellularlocation^b^	CorrespondingEST^c^
			aa	Mw(kDa)	pI		
***PvGF14n***	**Phvul.005G066600**	**Chr05:10288469–10295349 (+strand)**	**263**	**30.21**	**4.75**	**Cyto, chl, per**	**CV539470**
***PvGF14d***	**Phvul.005G095500**	**Chr05:28501915–28505383 (+strand)**	**261**	**29.56**	**4.71**	**Cyto, chl, per**	**GW892341**
***PvGF14c***	**Phvul.002G238300**	**Chr02:40393090..40396629 (-strand)**	**258**	**29.25**	**4.77**	**Cyto, chl, per**	**CV530328**
***PvGF14q***	**Phvul.002g102500**	**Chr02:20470454..20474678 (-strand)**	**258**	**29.45**	**4.75**	**Cyto, mito, per**	**none**
***PvGF14e***	**Phvul.003G043200**	**Chr03:4827912..4831544 (-strand)**	**259**	**29.51**	**4.83**	**Cyto, chl, per**	**HS103842**
***PvGF14b***	**Phvul.009G143400**	**Chr09:20974074..20976460 (-strand)**	**248**	**28.19**	**4.85**	**Cyto, chl, per**	**FE704892**
***PvGF14a***	**Phvul.008G004300**	**Chr08:482680..485463 (-strand)**	**256**	**28.98**	**4.59**	**Cyto, chl, per**	**CV530850**
***PvGF14g***	**Phvul.008G162500**	**Chr08:41741118..41743699 (+strand)**	**261**	**29.30**	**4.57**	**Cyto, chl, per**	**GW901700**
***PvGF14h***	**Phvul.009G032500**	**Chr09:7120999..7123241 (+strand)**	**259**	**29.16**	**4.56**	**Cyto, chl, per**	**GW904281**

^a^ Chromosomal location indicates the position of each gene in chromosome

^b^cyto, chl, mito and per refer to cytoplasm, chloroplast, mitochondrial and peroxisome, respectively

^c^EST (Expressed Sequence Tags) accession with the highest homology to corresponding *PvGF14* gene; aa, amino acid; pI, isoelectric point; Mw, molecular weight.

An alignment of deduced amino acid sequences of PvGF14s with each other indicated that the isoforms exhibit high sequence conservation with the identity ranging from 63.0% to 94.2% at amino acid level (Fig 1 and S4 Table). The sequence diversification mainly occurred at the N-terminal and the C-terminal regions, suggesting that those regions are possibly responsible for isoform specificity [47]. Additionally, sequence conservation at amino acid level (57.6% to 96.9%) was also observed between soybean and common bean 14-3-3 proteins. Each PvGF14 showed more than 92% sequence identity with its orthologs in soybean (S5 Table).

### Evolutionary relationship and gene structure of 14-3-3 gene family in common bean

**Fig 2:**
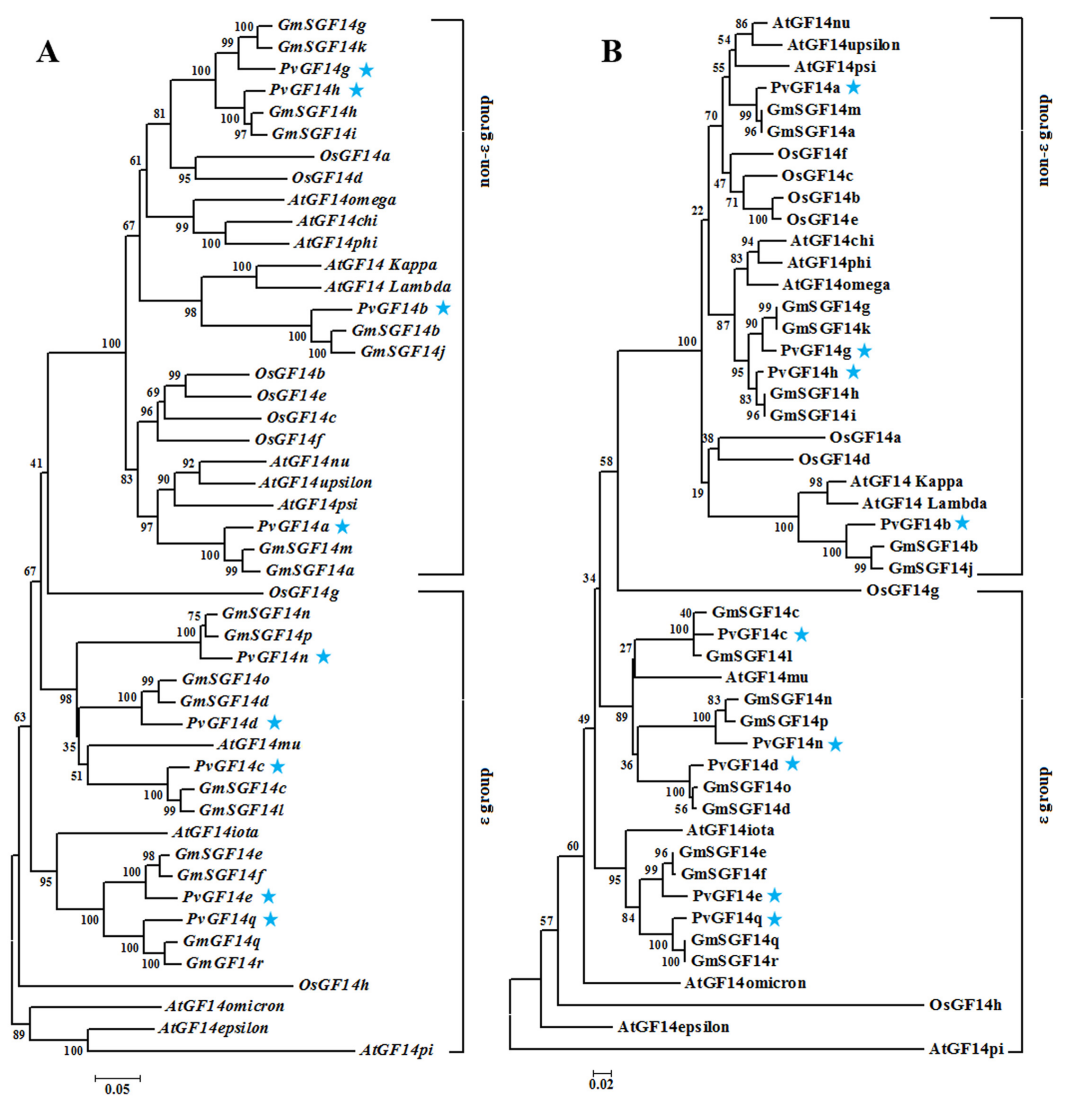
Phylogenetic analysis of PvGF14s and other GF14s from different species. Phylogenetic trees were calculated based on CDS matrix (A) and protein matrix (B) from common bean (PvGF14), soybean (GmSGF14), Arabidopsis (AtGF14) and rice (OsG14F), and the tree was classified into epsilon and non-epsilon groups. Each PvGF14/*PvGF14* is indicated by a star.

To examine the evolutionary relationship of 14-3-3 proteins from common bean and other plant species (soybean, Arabidopsis and rice), a phylogenetic analysis was conducted at both nucleotide and protein levels. In both the cases, trees with similar topologies were obtained (Fig 2). PvGF14n, PvGF14d, PvGF14c, PvGF14e and PvGF14q were clustered into the epsilon-like class, while PvGF14a, PvGF14g, PvGF14h and PvGF14b were grouped together with non-epsilon isoforms of 14-3-3 proteins from Arabidopsis, rice and soybean. Each PvGF14 was clustered together with two GmSGF14 orthologs (Fig 2 and S5 Table). The analysis revealed that PvGF14s are evolutionarily closer to soybean 14-3-3s compared to Arabidopsis and rice, which is consistent with the species evolutionary history [40]. The 14-3-3s from Arabidopsis and rice formed separate clades or branches in the phylogenetic tree such as AtGF14pi, AtGF14epsilon, AtGF14omicron and OsGF14h (Fig 2), suggesting that these epsilon-like 14-3-3 genes were lost in common bean and soybean during evolution or evolved a new function.

To better understand the evolutionary relationship between 14-3-3s, the ratios of Ka/Ks for 14-3-3 pairs from common bean, soybean, Arabidopsis and rice were estimated (S6 Table). As a result, the Ka/Ks values ranged from 0 to 0.347 with an average of 0.083.All the 14-3-3s appear to be under purifying selection during evolution, as their Ka/Ks ratios were estimated <1. Since each of *PvGF14s* and its two closely-related orthologs in soybean were clustered into same discrete clade in the phylogenetic tree, the Ka/Ks ratios were further observed. The Ka/Ks ratios for the closest ortholog pairs varied from 0 to 0.153 with an average of 0.060, suggesting that the ortholog pairs among legumes tend to have less evolutionary diversification.

**Fig 3:**
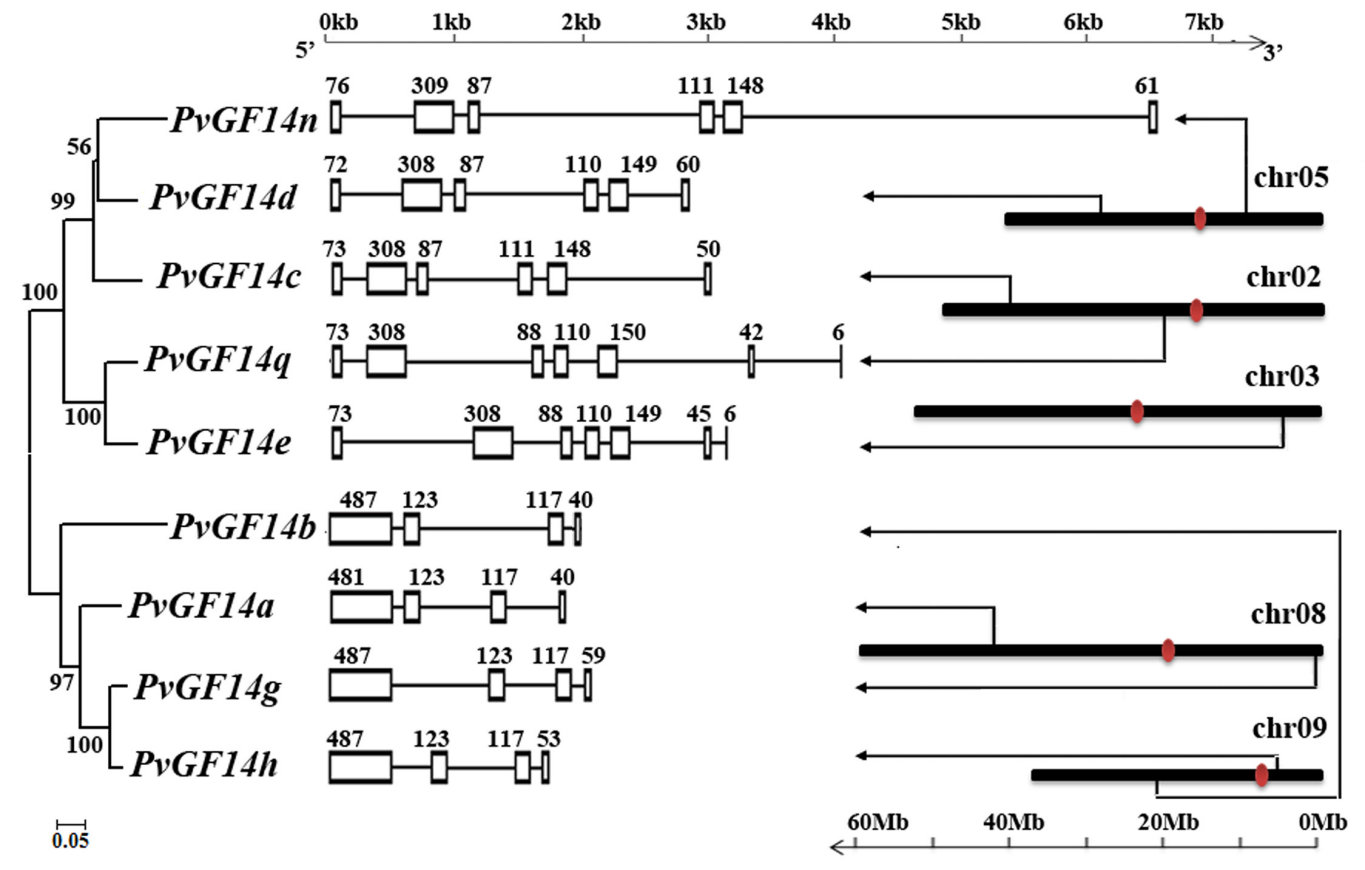
Gene structures and chromosomal localization of *PvGF14s* in common bean. The left panel is the phylogenetic tree of *PvGF14s*; the middle panel is the intron-exon structures where the exons are shown by rectangular, and the introns are represented by thin lines; the right panel displays the chromosomal localization of *PvGF14s*.

To investigate the exon-intron organization in *PvGF14s*, gene structures were mapped on the basis of the genomic and coding region sequences. Common bean 14-3-3gene structure comprised of 4 exons in non-epsilon class, and 6–7 exons in epsilon-like class (Fig 3). The *PvGF14s* belonging to the same class contained similar size of exons, such as *PvGF14q* and *PvGF14e*, *PvGF14n* and *PvGF14d*, *PvGF14g* and *PvGF14h* (Figs 2 and 3). Evidently, conserved gene structure of *PvGF14s* strongly supports the reliability of phylogenetic tree.

### Chromosomal distribution and duplications of *14-3-3s* in common bean

The nine *PvGF14s* are located on five different chromosomes (chromosomes 2, 3, 5, 8 and 9) in common bean. Each chromosome contains two *PvGF14s* except chromosome 3 (Fig 3). Gene family can arise from the segmental duplication or tandem amplification of chromosomal regions [48]. Generally, tandem amplification was defined as two paralogs separated by less than five genes in the same chromosome. The *PvGF14s* in the same chromosome were distributed far from each other (Table 1 and Fig 3), suggesting that common bean 14-3-3 gene family was likely derived from segmental duplication rather than tandem amplification of chromosomal regions. Furthermore, we investigated whether traceable genome duplications contributeto the expansion of the 14-3-3gene familyin common bean. The results revealed that the sets of *PvGF14s* (*PvGF14c* and *PvGF14d*, *PvGF14q* and *PvGF14e*) were mapped on the duplicated block120 and block93, respectively, suggesting that these two pairs were possibly derived from segmental duplication events during the evolutionary process. No traceable duplication event was observed for other *PvGF14s*. To investigate the selective evolutionary pressure on *PvGF14* gene divergence after duplication, the non-synonymous/synonymous substitution ratio (Ka/Ks) was retrieved for the two duplication pairs of 14-3-3 genes. Consequently, Ka/Ks value of the gene duplication pairs, *PvGF14c* and *PvGF14d* as well as *PvGF14q* and *PvGF14e* were 0.058 and 0.073, respectively, suggesting that these genes possibly have undergone a purifying selection with limited functional divergence after duplication.

### Expression analysis of *PvGF14s* in common bean tissues

**Fig 4:**
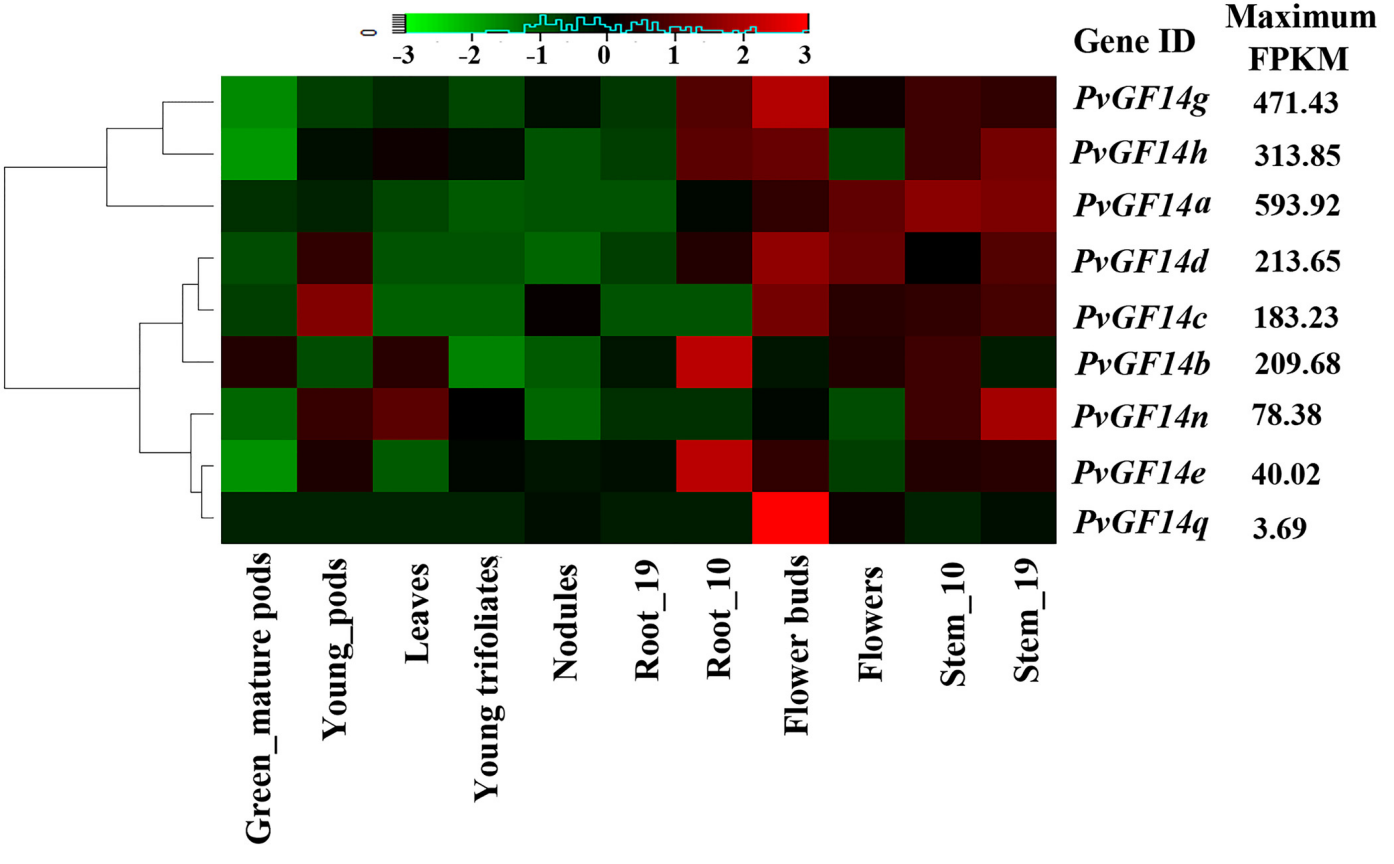
Expression analysis of *PvGF14* genes in various tissues. The transcriptome data of common bean across different tissues and developmental stages were extracted from the publicly-available Phytozome database (http://www.phytozome.net) for heatmap generation. The color scale above the heat map indicates gene expression levels, low transcript abundance indicated by green color and high transcript abundance indicated by red color. Maximum FPKM value for each *PvGF14* is shown.

To investigate expression patterns of *PvGF14s*, we utilized the publicly available genome-wide transcript profiling data of common bean tissues from Phytozome database (http://www.phytozome.net), which contains RNAseq reads from vegetative tissues (trifoliates, nodule, root, stem, leaf) and productive tissues (flower bud, flower, pod). All the common bean 14-3-3 genes showed tissue-specific expression patterns (S7 Table). Over all, *PvGF14* transcripts can be categorized into 3 groups based on their expression patterns (Fig 4). Group 1 comprised of *PvGF14g*, *PvGF14h* and *PvGF14a*, which were mainly expressed in stems, flower buds and/or root_10. Group 2 contained *PvGF14d*, *PvGF14c* and *PvGF14b* with high expression in flowers, flower buds, stems and/or pods, while group 3 consisted of *PvGF14n*, *PvGF14e* and *PvGF14q* with transcript abundance either in stems or roots or flower buds. Also, four representative *PvGF14s* (*PvGF14c* and *PvGF14d* from epsilon-like class, *PvGF14a* and *PvGF14b* from non-epsilon class), were chosen to confirm the publicly available transcript profiling data using RT-PCR approach, and similar expression patterns were observed in stem, leaf, flower and pod of common bean (S1 Fig).

The maximum fragments per kilo base of transcript per million mapped reads (FPKM) for *PvGF14q* was low (3.69) compared to the reads for other *PvGF14s* (40.02 to 593.92). Additionally, blast search against Expressed Sequence Tags (ESTs) in NCBI database did not find any EST corresponding to *PvGF14q* (Table 1). Our attempts to amplify *PvGF14s* using cDNA synthesized from RNA isolated from several different tissues of common bean yielded successful results for all the *PvGF14s* except *PvGF14q*. These results indicated that common bean contain only 8 putative 14-3-3 genes that are transcribed.

### Effect of abiotic stresses on the expression of *PvGF14s*

Several studies have documented a role of plant 14-3-3 proteins in abiotic stress response [24–31]. To investigate if PvGF14s also have similar roles in common bean, we examined the expression patterns of *PvGF14s* in response to cold, drought and salinity stress.

**Fig 5:**
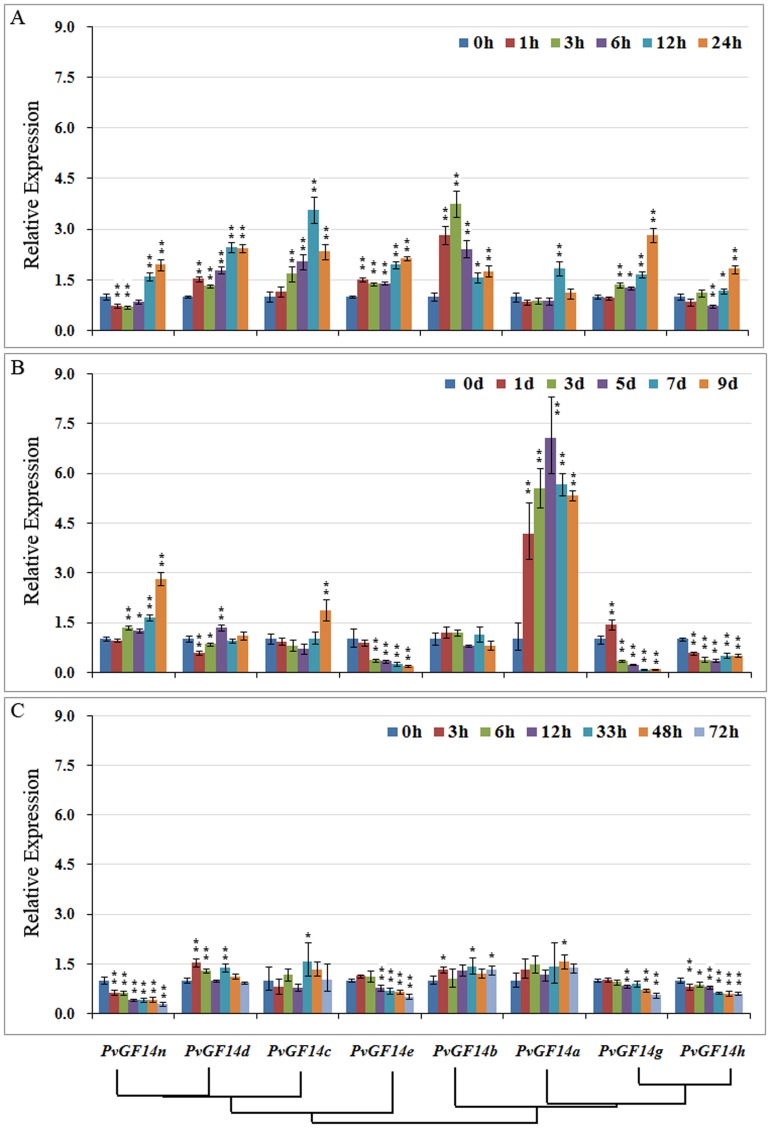
Expression analysis of *PvGF14* genes in response to abiotic stresses. Ten-day-old common bean seedlings were exposed to stress treatment as indicated below. Gene expression analysis was conducted by qRT-PCR using gene specific primers. (A) gene expression pattern of *PvGF14s* in seedlings exposed to cold stress for 0, 1, 3,6, 12 and 24h. (B) gene expression pattern of *PvGF14s* in seedlings exposed to drought stress for 0, 1, 3, 5, 7 and 9d. (C)gene expression pattern of *PvGF14s* in seedlings exposed to salinity stress for 0, 3, 6, 12, 33, 48 and 72h. Error bars indicate SE of two biological and three technical replicates. Values were normalized against the *ACTIN11* gene. Significant differences are denoted by asterisks: * p<0.05,**p< 0.01.

Ten-day-old common bean seedlings were exposed to cold stress at 4°C for 0, 1, 3, 6, 12 or 24h, and expression of *PvGF14*s were monitored. The results revealed that cold stress altered the expressions of *PvGF14s* that could be grouped into 2 categories. As indicated in Fig 5A, category 1 contained genes that showed gradual increase in transcript accumulation as the stress prolonged. For example,*PvGF14n*, *PvGF14d*, *PvGF14e*, *PvGF14g* and *PvGF14h* transcript levels increased to 1.9, 2.1, 2.6, 2.8 and 1.8 fold, respectively as compared to control. All these five gene family members were expressed to their highest level either at 12 or 24h after cold stress. The category 2 comprised the genes (*PvGF14a*, *PvGF14b* and*PvGF14c*) whose transcript levels increased with cold stress treatment followed by gradual decrease as the stress continued. *PvGF14c* and *PvGF14a* transcripts reached to their maximum level at 12h cold stress with 3.58 and 1.85 fold increase, respectively as compared to control, while *PvGF14b* was expressed to the highest level at 3h with 3.73 fold increase as compared to control.

When common bean seedlings were exposed to drought stress, the expression of *PvGF14e*, *PvGF14g*and*PvGF14h* was decreased to 5.3, 13.0 and 2.9 fold on 9, 7 and 5 days after treatment, respectively (Fig 5B). On the contrary, a distinct increase in transcript accumulation on 9 days of drought treatment was observed for *PvGF14n* and *PvGF14c* with 2.82 and 1.86 fold changes, respectively. A dramatic increase in *PvGF14a* transcript (5.32 fold) as compared to control was observed on 5 days of drought treatment.

When young seedlings were subjected to salt stress, the expressions of *PvGF14n*, *PvGF14e*, *PvGF14g* and *PvGF14h* were gradually decreased as the salt stress duration increased, and their maximum fold changes were up to 3.54, 1.97, 1.83 and 1.67, respectively (Fig 5C). On the contrary, the expressions of *PvGF14d*, *PvGF14b* and *PvGF14a* were pronounced at one or more stress time points by 1.54, 1.42 and 1.47 fold, respectively as compared to non-salinity control. *PvGF14q* transcript was not detected for any of the stress treatment used in this study.

To better understand the role of PvGF14s in abiotic stress response, promoter analysis was also conducted. It was predicted that promoter regions of *PvGF14e* and *PvGF14g* contain LTR *cis*-acting element involved in low-temperature responsiveness, supporting 2.6 and 2.8fold increase in the expression of *PvGF14e* and *PvGF14g* under cold stress. *PvGF14q*, *PvGF14n* and *PvGF14d* contain ABRE *cis*-acting element in their promoter, which is implicated in ABA response. HSE, a *cis*-acting element related to heat stress response, was found in the promoters of *PvGF14q*, *PvGF14c*, *PvGF14n*, *PvGF14g* and *PvGF14h*. All the 14-3-3 genes except *PvGF14c* contain TC-rich repeat, a *cis*-acting element involved in defense and stress response. In addition, a MYB binding site associated with drought-inducibility, was predicted in the promoter region of all the *PvGF14s*. The prediction of promoter elements provided some clues for the response of *PvGF14s* to various abiotic stresses.

### PvGF14 proteins form dimers

**Fig 6:**
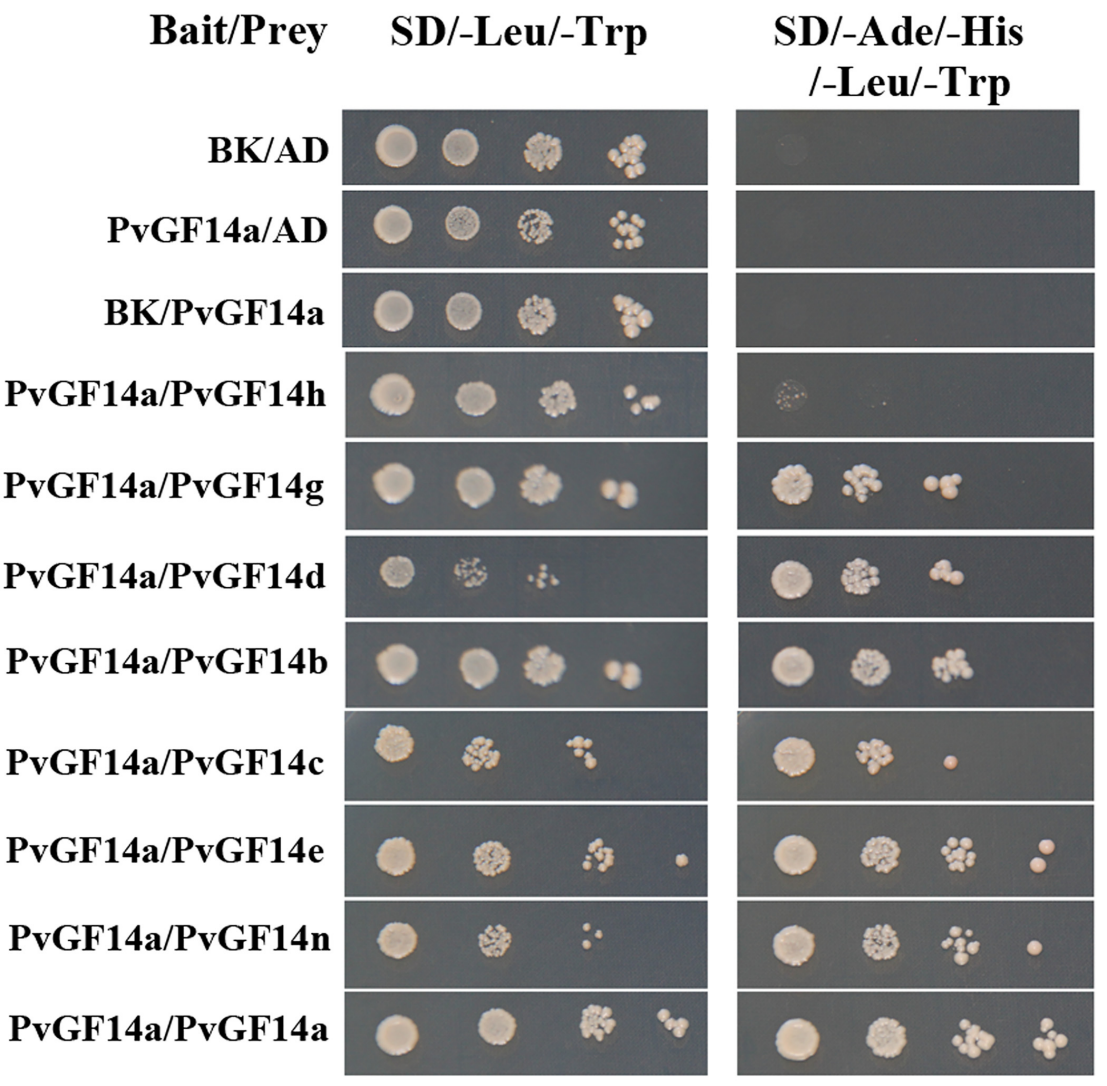
Interaction between PvGF14a and other PvGFs in yeast two-hybrid assay. Yeast cells were co-transformed with combination of DNA-binding domain (BK, Bait) and activation domain (AD, Prey) fused constructs as indicated. Yeast suspension culture (5μL) with a series of 10X dilutions was spotted onto synthetic defined (SD) selection plates. Growth on SD without leucine and tryptophan (SD/-Leu/-Trp) showed the presence of both the vectors, while growth on SD without leucine, tryptophan, adenine and histidine (SD/-Ade/-His/-Leu/-Trp) indicated interaction between bait and prey.

It has been established that 14-3-3 isoforms function as homo- or heterodimers creating 14-3-3 isoform specificity [3,49]. To investigate whether PvGF14s also form dimers, we chose *PvGF14a* as a representative and investigated its ability to form dimer with itself and with other PvGF14 isoforms. *PvGF14a*was chosen because it not only showed an increased transcript abundance in response to cold, drought and salinity stress (Fig 5), but also exhibited the highest FPKM value among the *PvGF14s* (Fig 4). As shown in Fig 6, co-transformed yeast colonies containing PvGF14a (bait) and other PvGF14s (prey) grew on SD/-Ade/-His/-Leu/-Trp, while yeast colony in three negative controls including bait/prey empty vectors, PvGF14a/empty prey vector and empty bait vector/PvGF14a did not grow on SD/-Ade/-His/-Leu/-Trp, indicating that PvGF14a interacts with each of all the examined PvGF14s in yeast cell, including itself. This result was consistent with previous report that 14-3-3ω in Arabidopsis could form homodimers as well as heterodimers with multiple isoforms [50]. Although the interaction activity was not quantified in the study, a weak interaction was observed between PvGF14a and PvGF14h, supporting the fact that preferences for certain dimer combinations exist among 14-3-3 isoforms [3].

## Discussion

14-3-3 proteins are implicated in a wide range of cellular and physiological processes in plants and other eukaryotes. The functional diversity and specificity of 14-3-3 isoforms have been studied in great detail [2,51]. Even though a large number of 14-3-3s have been identified in several plant species, additional 14-3-3s are expected to be identified and characterized in more plant species, especially those with economic importance for elucidating their roles in developmental processes or stresses. In this study, we identified 9 14-3-3 isoforms (PvGF14s) in common bean. Similar to soybean and other plant species, they are grouped into epsilon and non-epsilon groups with distinct intron-exon structures (Figs 2 and 3). Phylogenetic analysis, Ka/Ks ratios and sequence identity suggested that *PvGF14s* share closer evolutionary relationship with GmSGF14s from soybean and that the 14-3-3s from these two plant species may share similar function. They not only formed a discrete clade in the phylogenetic tree (such as *PvGF14g*, *GmSGF14g* and *GmSGF14k*), but also showed low Ka/Ks ratios (0–0.153) and above 92% sequence identity (Fig 2, S5 and S6 Tables). This result was not surprising since both soybean and common bean belong to the *leguminosae* family and had undergone whole-genome duplication event ~56.5 million years ago [52]. Intriguingly, the number of 14-3-3s identified in common bean is only half of the ones in soybean, and each PvGF14 corresponds to two SGF14s orthologs in soybean with sequence identity above 92% (S5 Table). These observations are consistent with the evolutionary history that common bean and soybean diverged ~19.2 million years ago, and soybean subsequently experienced another whole-genome duplication event independently of common bean [53]. The evolutionary relationship and sequence identity together with genome evolution suggest that each PvGF14 possibly share similar function with soybean 14-3-3s belonging to the same clade. Additionally, phylogenetic analysis indicated that *PvGF14q* was clustered together with *GmSGF14q* and *GmSGF14r* (Fig 2), which are not transcribed in soybean [14]. Our attempts to amplify *PvGF14q* from different tissues of common bean under normal and stress conditions failed to detect any transcript, suggesting that *PvGF14q* is possibly a pseudogene or transcribed at specific developmental processes or under special conditions. In this study, 8*PvGF14s* were successfully cloned, and their transcribed sequences were identical to the prediction obtained from Phytozome database, thus verifying the gene organizations of these 8*PvGF14s* (Fig 3). Furthermore, our yeast two-hybrid assay also indicated that these 8 PvGF14s form active proteins with functional protein-protein interaction domains (Fig 6).

Stress induced 14-3-3 isoforms have been reported in many plant species such as Arabidopsis, rice, tomato, maize, cotton and *Physcomitrella patens* [26,54–60]. Over-expression of *14-3-3* isoforms can increase or reduce stress tolerance in cotton, Arabidopsis, maize and rice [24,25,55], indicating that 14-3-3 family plays regulatory roles in response to stress. The presence of stress-responsive elements in the promoter regions of *PvGF14s* pointed out their possible roles in response to cold, drought and salinity stress. Furthermore, the altered expression patterns of *PvGF14* genes in response to cold, drought and salinity (Fig 5) suggested their prominent roles under these stresses. Some common bean *PvGF14s* displayed similar expression pattern to their homologs in other plant species under cold, drought and salinity stress. For example, *RCI1A/RCI1/14-3-3ψ* and *RCI1B/RCI2/14-3-3λ* in Arabidopsis are most closely related to *PvGF14a* and *PvGF14b* within the phylogenetic tree (Fig 2). *RCI1A* and *RCI1B* are two cold-inducible genes that are involved in freezing tolerance and cold acclimation in Arabidopsis [27,30]. The expression of *PvGF14a* and *PvGF14b* were elevated 1.85 and 3.73 fold, respectively by cold stress (Fig 5A), implying that *PvGF14a* and *PvGF14b* possibly function as a modulator of cold-induced signaling pathways. Similar to *TFT1* and *TFT4* in tomato that were up-regulated by salt treatments [29], we found that transcript accumulation of *PvGF14b* (homolog of *TFT1* and *TFT4* in common bean) increased by 1.42 fold under salt stress (Fig 5C). Likewise, the expression of *ZmGF14-6* in maize was down-regulated by drought stress [55], and transcript level of its closely-related homolog in common bean (*PvGF14h*) decreased by 2.9 fold under drought (Figs 2 and 5B). These similarities in the expression patterns suggest that they might perform similar functions as their homologs in other plant species.

It has been well accepted that plant responses and signaling pathways activated by stresses are largely overlapping. Sun et al. (2011) reported that *CGF14-4* was more sensitive to both drought and salinity stress, while other *14-3-3s* in cotton responded only to either drought or salinity stress [54]. The evidence from tomato indicated that *TFT7*, a tomato *14-3-3* gene, mediates crosstalk between salt stress and potassium and iron-deficiency signaling pathways in roots [56]. In the study, *PvGF14n*, *PvGF14c* and *PvGF14a* were up-regulated (1.9–5.3 fold) after exposure to cold and drought stress, while *PvGF14e*, *PvGF14g* and *PvGF14h* were down-regulated (1.7–13.0 fold) by both salt and drought stress (Fig 5), suggesting that these 14-3-3 genes may play a role in crosstalk between drought and salinity or cold stress signaling pathways. However, some *PvGF14s* can differentially respond to these abiotic stresses. For example, the expressions of *PvGF14e* and *PvGF14h* were increased by cold stress and decreased by salinity and drought stress (Fig 5), consistent with the previous report that *ZmGF14-6* was activated in response to salinity and depressed by drought stress [55]. These observations suggested that *PvGF14s* might perform functions in a stress-specific manner.

Spatial-temporal or specific expression of *14-3-3* isoforms is a crucial determinant of isoform specificity [14,29,30,61,62]. The differential expression pattern of *PvGF14s* in various tissues indicated their organ-specific functions (Fig 4). When exposed to cold, drought and salinity stress, expression pattern of *14-3-3* isoforms differed among the same group, and even different expression change occurred among the most closely relevant *14-3-3* isoforms (Fig 5). Thus, the organ-specific and stress-specific properties of *PvGF14s* provided important clues for elucidating their functions and isoform specificity. It has been proposed that members of a certain evolutionary branch have potential to share similar interactions and functions [3]. PvGF14h and PvGF14g are the closest isoform pair in the phylogenetic tree (Fig 3), and they also showed 94.2% sequence identity (S4 Table). Noticeably, similar expression patterns were observed for *PvGF14h* and *PvGF14g* in response to cold, drought and salinity stresses (Fig 5) and indifferent tissues during the development (Fig 4). The observations suggest that *PvGF14h* and *PvGF14g* perform similar cellular functions during developmental processes or in response to various stresses.

In conclusion, this study presents a comprehensive classification of common bean 14-3-3s. Although there are 9 predicted *PvGF14s* in common bean, only 8 are transcribed. The detail characterization of *PvGF14s* in terms of their transcript accumulation in different tissues during the development and in response to a variety of abiotic stresses provides strong evidence for isoform specificity in common bean. This research outcome adds new members into plant 14-3-3 family, and also strengthens the link between 14-3-3s and stress response. Future study will aim at investigating the effect of each *PvGF14* gene on stress tolerance, identifying their potential clients and functional interaction, and 14-3-3 isoform combination in response to specific stress.

## Supporting Information

S1 Fig RT-PCR analysis of expression pattern of *PvGF14s* in stem, leaf, flower and pod of common bean. (TIF)

S1 Table The accession numbers of GF14 proteins used in the study. (XLSX)

S2 Table Primers used for gene cloning. (XLSX)

S3 Table Primers used for qPCR. (XLSX)

S4 Table Identity of PvGF14s with each other. (XLSX)

S5 Table Identity of PvGF14s with GF14s from soybean. (XLSX)

S6 Table The Ka/Ks calculation of 14-3-3 pairs from common bean, soybean, Arabidopsis and rice. (XLSX)

S7 Table FPKM of *PvGF14s* in different tissues. (XLSX)
