# Supplementary figures and images for: Identification of 14-3-3 Family in Common Bean and Their Response to Abiotic Stress
Source: PLoS One. 2015 Nov 23;10(11):e0143280. doi: 10.1371/journal.pone.0143280 (PMC4658069; doi:10.1371/journal.pone.0143280)

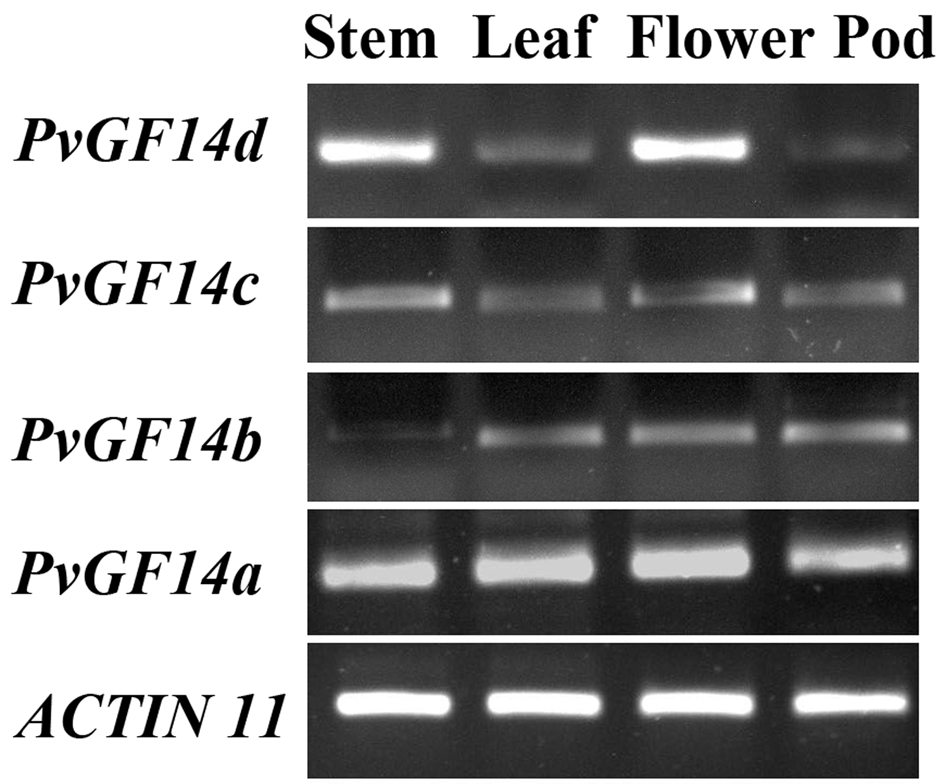

Supplement: S1 Fig — (TIF) [file pone.0143280.s001.tif]
